# Tumor‐proximal liquid biopsy to improve diagnostic and prognostic performances of circulating tumor cells

**DOI:** 10.1002/1878-0261.12534

**Published:** 2019-07-25

**Authors:** Etienne Buscail, Laurence Chiche, Christophe Laurent, Véronique Vendrely, Quentin Denost, Jérôme Denis, Matthieu Thumerel, Jean‐Marc Lacorte, Aurélie Bedel, François Moreau‐Gaudry, Sandrine Dabernat, Catherine Alix‐Panabières

**Affiliations:** ^1^ INSERM U1035 Bordeaux France; ^2^ CHU de Bordeaux France; ^3^ Université de Bordeaux France; ^4^ Laboratory of Rare Human Circulating Cells University Medical Centre of Montpellier France; ^5^ Service de Biochimie Endocrinienne et Oncologie Hôpital Pitié Salpêtrière Assistance Publique Hôpitaux de Paris France

**Keywords:** cancer diagnostics, cancer prognosis, circulating tumor cells, liquid biopsy, vascular organ drainage

## Abstract

Circulating tumor cell (CTC) detection and numeration are becoming part of the common clinical practice, especially for breast, colon, and prostate cancer. However, their paucity in peripheral blood samples is an obstacle for their identification. Several groups have tried to improve CTC recovery rate by developing highly sensitive cellular and molecular detection methods. However, CTCs are still difficult to detect in peripheral blood. Therefore, their recovery rate could be increased by obtaining blood samples from vessels close to the drainage territories of the invaded organ, when the anatomical situation is favorable. This approach has been tested mostly during tumor resection surgery, when the vessels nearest to the tumor are easily accessible. Moreover, radiological (including echo‐guided based and endovascular techniques) and/or endoscopic routes could be utilized to obtain CTC samples close to the tumor in a less invasive way than conventional biopsies. The purpose of this article is to summarize the available knowledge on CTC recovery from blood samples collected close to the tumor (i.e., in vessels located in the drainage area of the primary tumor or metastases). The relevance of such an approach for diagnostic and prognostic evaluations will be discussed, particularly for pancreatic ductal adenocarcinoma, colorectal adenocarcinoma, hepatocellular carcinoma, and non‐small‐cell lung cancer.

**Abbreviations**
CRC: colorectal cancer
CTC: circulating tumor cell
CT: computed tomography
EMT: epithelial‐to‐mesenchymal transition
EUS‐FNA: endoscopic ultrasound‐guided fine‐needle aspiration
FNAC: fine‐needle aspiration cytology
HCC: hepatocholangiocarcinoma
MRI: magnetic emission imaging
NSCLC: non‐small‐cell lung cancer
OS: overall survival
PCR: polymerase chain reaction
PDAC: pancreatic ductal adenocarcinoma
PET: positron emission tomography
PFS: progression‐free survival
RT‐qPCR: reverse transcription polymerase chain reaction

## 1 Introduction

Cancer diagnosis usually relies on information obtained using sequential procedures, including imaging data (CT, PET, MRI, ultrasonography, X‐rays), changes in the levels of markers in bodily fluids (e.g., blood, urine), and mainly on the pathology examination of cancer cell or tissue samples, obtained by surgical biopsy or by fine‐needle aspiration (fine‐needle aspiration cytology, FNAC). Biopsy and FNAC are invasive procedures, especially in the case of deeply located tumors, and may present severe complications such as infection, bleeding, or inflammation. More importantly, they also carry the risk of seeding tumor cells around the sampling area. Indeed, detached cells can be cleared by interstitial fluids to lymph nodes, or into the veins draining the tissue, thus entering the circulation. They might then extravasate at distant healthy tissues and contribute to metastasis formation. During fine‐needle aspiration, cells can be dragged along the needle track, leading to the possibility of increasing the local dissemination (Shyamala *et al*., 2014). Moreover, if the fraction of tumor cells in the biopsy is too low for pathology/molecular analyses, particularly in tumors with strong desmoplastic reaction, repeated sampling is required, possibly delaying tumor management.

Besides diagnosis, cancer management would highly benefit from broadening the panel of the available prognostic/predictive markers to better stratify patients in view of precision medicine, and to follow the tumor response after treatment initiation. In particular, the outcome of pancreatic cancer is highly unpredictable, even in the case of resectable tumors, because predictive and prognosis markers are missing (Zhou *et al*., 2017). Consequently, effective and reliable biomarkers need to be identified for rapid diagnosis, especially when pathology‐based diagnosis is not available or noncontributive.

Primary tumors and metastases release in the blood and other body fluids tumor‐derived elements, such as circulating tumor cells (CTCs), nucleic acids, exosomes, and proteins. When identified as tumor‐derived, these elements can be considered as an evidence of the presence of a tumor (Alix‐Panabières and Pantel, 2014). The analysis of these circulating tumor‐derived elements, called ‘liquid biopsy’ (Alix‐Panabières and Pantel, 2013; Pantel and Alix‐Panabières, 2010), might represent a noninvasive, safer, and faster alternative/complement to tissue biopsy. Tumor elements are released very early during cancer development. For example, in a mouse model of pancreatic cancer, CTCs with metastatic potential are already shed during the formation of the primary pancreatic adenocarcinoma, before it becomes detectable by histologic methods (Rhim *et al*., 2012). Liquid biopsies can also be used to detect disease progression or treatment resistance before the appearance of the first clinical signs (Riethdorf *et al*., 2018).

The first CTC proof was published in 1869 by Thomas Ashworth (1869). From the 1970s, the interest on CTCs has gradually increased thanks to the progress in the detection methods based on molecular biology techniques. In the last 20 years, new technologies for CTC enrichment, detection, and characterization with higher sensitivity have been developed, allowing CTC numeration in different solid cancers (Lianidou *et al*., 2014). For instance, the US Food and Drug Administration (FDA) has approved the use of the CellSearch^®^ test for CTC detection in patients in the clinical routine for metastatic breast cancer in January 2004 (Cristofanilli *et al*., 2004), and for the prognosis of advanced colorectal and prostate cancer in November 2007 (Cohen *et al*., 2006, 2009) and February 2008 (Resel *et al*., 2010), respectively (Millner *et al*., 2013; Riethdorf *et al*., 2018). Since then, increasing evidence indicates that CTC detection is a very promising tool, mostly of prognostic value in lung cancer, especially non‐small‐cell lung cancer (NSCLC), colorectal cancer (CRC), hepatocholangiocarcinoma (HCC), and pancreatic ductal adenocarcinoma (PDAC) (Hench *et al*., 2018; Pimienta *et al*., 2017).

Most studies have focused on CTC detection and counting in peripheral blood samples obtained by puncture of the median cubital vein. Fewer reports have tested the hypothesis that the chances of capturing and detecting CTCs might be higher in vessels closer to the tumor, especially in the main veins that drain blood from the organ invaded by the cancer. In this review, we will discuss studies that compared CTC yields in peripheral blood and in blood from vessels in the vicinity of the primary tumor (NSCLC, CRC, HCC, and PDAC). As blood sampling in the main vessels close to the tumors is feasible in the early management of cancer or during surgery, we focused on the diagnostic and prognostic values of this approach and considered the possible added value of the ‘close‐to‐the‐tumor liquid biopsy’.

## 2 Basis for CTC analysis in the main veins close to the tumor site

The primary tumor releases a heterogeneous population of circulating cells, such as cells with metastatic potential, apoptotic or necrotic cells that are cleared by the organism, and live cells that can remain in a latent or dormant state in a distant organ (Massagué and Obenauf, 2016; Nguyen *et al*., 2009).

CTCs are disseminated mostly during metastasis formation. In fact, very few of the tumor cells released into the circulation will form metastases (Kessenbrock *et al*., 2010; Luzzi *et al*., 1998; Martin *et al*., 2017; Massagué and Obenauf, 2016). First, epithelial tumor cells in the primary tumor undergo a reversible phenotypic change, known as epithelial‐to‐mesenchymal transition (EMT). Consequently, cells detach from the tumor and spread out, using the surrounding fluids to move away, and enter the vessels by extravasation (Tam and Weinberg, 2013; Thiery *et al*., 2009). The first capillary bed that a metastatic cell encounters depends on the blood circulation pattern near the primary tumor. In most organs, the venous circulation leads to the right ventricle of the heart and into the lungs, whereas the gut venous circulation drains into the liver. This explains the high incidence of metastases in lungs and liver (Denève *et al*., 2013; Nguyen *et al*., 2009). For this reason, some authors have used blood samples from the vena cava upstream of the liver for CTC detection in patients with metastatic breast cancer (samples were obtained from an implanted vascular device) (Peeters *et al*., 2011).

## 3 Technologies for CTC enrichment and detection

Although the release of tumor cells from the primary tumor and/or metastases is deleterious for the patient, it also becomes an opportunity to obtain relevant information for precision medicine using a noninvasive procedure. Several technologies allow CTC enrichment and numeration (Alix‐Panabières and Pantel, 2014). As CTCs are rare events, a first enrichment step is required to allow their detection. Specifically, CTCs’ physical properties (i.e., size, deformability, density, and electrical charges) can be used to differentially enrich them from the numerous surrounding cells present in blood (Alix‐Panabières and Pantel, 2014; Harouaka *et al*., 2013; Pantel and Alix‐Panabières, 2010). CTCs can also be enriched and detected on the basis of their biological properties. For instance, positive selection‐based capture relies on the expression of tumor cell surface markers (most commonly EpCAM). This can be combined with the presence of epithelial‐specific intracytoplasmic proteins (such as cytokeratin 19, CK19) and the absence of the blood‐specific cell surface marker CD45. These features are the basis of the CellSearch^®^ system. On the other hand, negative selection‐based capture is an unbiased CTC enrichment step to eliminate the unwanted white blood cells. Antibodies against cell surface markers of the different blood cell types are used to pull down white blood cells, leaving the remaining supernatant enriched in CD45^(‐)^ endothelial cells and CTCs. After enrichment, the detection step is needed to confirm the presence of CTCs in the sample (Alvarez Cubero *et al*., 2017). This can be done using (a) immunocytological technologies (anti‐epithelial antibodies), (b) molecular (RNA‐based) technologies (e.g., RT‐qPCR for epithelial mRNA), and (c) functional assays (e.g., EPISPOT assay that detects only viable CTCs) (Alix‐Panabières and Pantel, 2014).

Despite improvements in the methods for CTC enrichment and detection, these cells remain rare in blood samples and difficult to identify. To maximize the chances of CTC recovery, it would seem logical to draw blood close to the site of the tumor. In the case of CRC, HCC, and PDAC, the primary tumor is connected to the vascular draining territory of the mesenteric and portal venous system, whereas lung cancer is linked to the pulmonary vein. These vessels are sufficiently large and resistant to allow direct vein puncture. Of note, the portal vein can be accessed by noninvasive ultrasonography puncture (Chapman and Waxman, 2016). The pulmonary vein is reachable only during surgery, but it is a good candidate to capture more CTCs, with a high prognostic value (Hashimoto *et al*., 2014). Conversely, in breast cancer and prostate cancer, tumor elements are released mostly in the lymphatic network and the internal iliac vasculature, respectively. As these draining systems cannot be punctured, CTC capture closer to the tumor has not been assessed in these cancer types. Therefore, this review will focus on CTC detection in the draining vessels of primary HCC, PDAC, CRC, and NSCLC.

## 4 Pancreatic ductal adenocarcinoma

**Figure 1:**
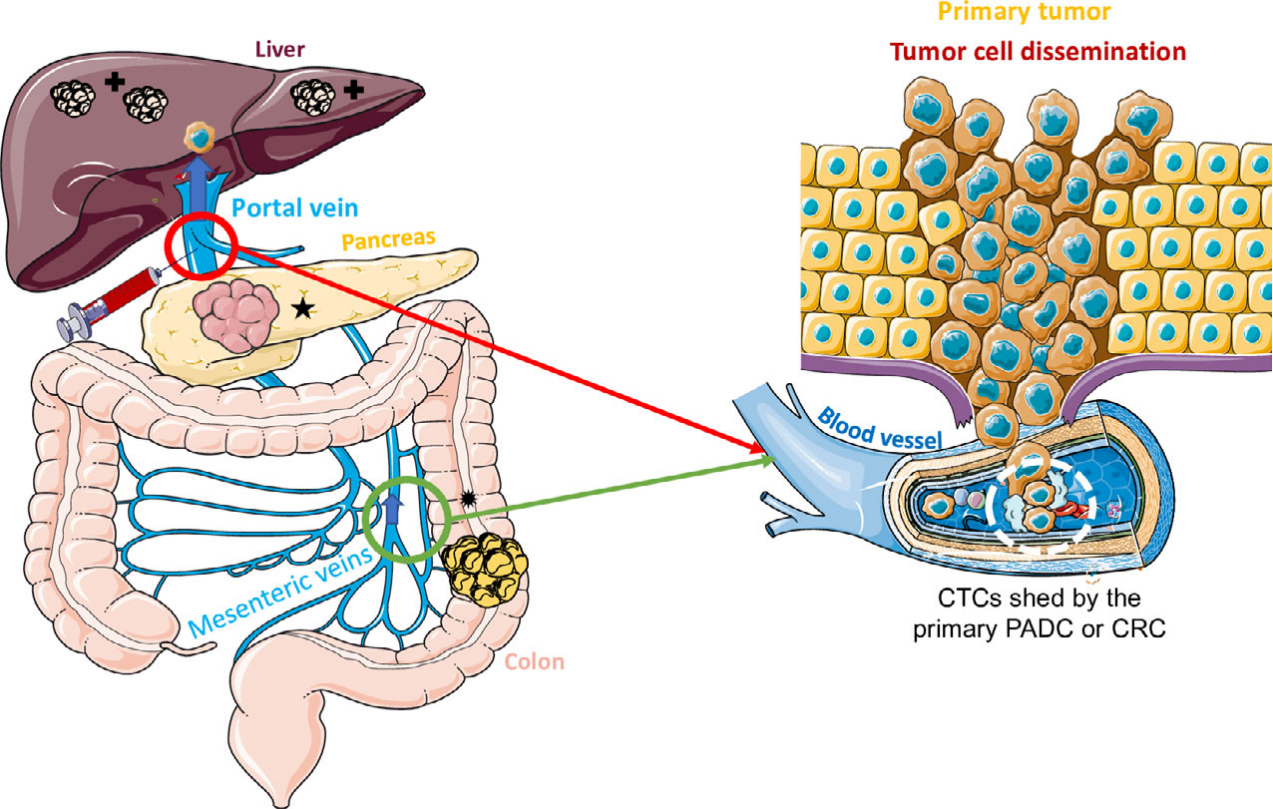
CTC detection in the portal vein for patients with PDAC (★) or CRC (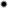). Pancreatic cancer and colorectal cancer metastases in the liver (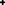) develop through multiple steps. Local invasion by cancer cells is followed by their intravasation into the tumor vasculature. Cancer cells then enter the porto‐mesenteric venous system as single cells or clusters that might be coated by platelets. CTCs are released in the superior and inferior mesenteric (green circle) veins for CRC in the right colon and left colon/rectum, respectively, and in the portal vein (red circle) for PDAC. Portal blood flows through the liver and then to other distant organs, after crossing the liver capillaries in portal areas. CTCs follow the same route and might extravasate in the liver parenchyma to start colonization. Portal blood sampling before passage in the liver can allow improving CTC recovery rate. The blue arrows show the direction of the blood flow in the veins.

Pancreatic ductal adenocarcinoma remains one of the deadliest cancers due to its late diagnosis and poorly efficient therapies (Buscail, 2017). Moreover, treatment is often delayed due to difficulties in proving the presence of malignant lesions (McGuigan *et al*., 2018). CTC numeration in the peripheral blood of patients with PDAC has been assessed as a diagnostic option, but rather unsuccessfully due to the low detection rates (Table S1). Most of these cohorts were quite small, and included only patients with metastatic or locally advanced tumors. Even in the cohorts that included patients with all tumor stages, metastatic cancers were the most frequent (>80%) (Lianidou *et al*., 2014). PCR‐based or physical‐based methods only slightly improved CTC detection rate (Table S1), whereas methods based on the expression of epithelial cell markers, such as CK19 or EpCAM, could have missed CTCs undergoing EMT. The most common site of PDAC spreading is the liver because the pancreas venous blood drains first into this organ (Figure 1) (Denève *et al*., 2013; Nieto *et al*., 2008). The liver filters pancreatic CTCs. If they do not stay in the liver, they will become highly diluted in the peripheral blood system (i.e., 1 tumor cell per 1 × 10^9^ blood cells, explaining the low detection rates) (Yu *et al*., 2011). To increase the chances of CTC detection, blood was sampled directly from the portal vein prior to CTC sequestration in the liver (Chapman and Waxman, 2016). This approach was first tested in 20 patients with resectable PDAC in whom portal blood could be easily and safely sampled during surgery (Bissolati *et al*., 2015) (Table 1). CTCs were detected (CellSearch^®^) in nine portal blood samples (45%) and in four peripheral blood samples (20%) from these patients. In 25% of the 20 patients, CTCs were detected only in the portal blood sample, and would have been missed if only peripheral blood was used. The presence of CTCs in peripheral or portal blood did not correlate with long‐term overall survival (OS) or progression‐free survival (PFS). Conversely, CTC detection in the portal vein sample was associated with higher rate of liver metastases (Bissolati *et al*., 2015). Another study compared CTC identification in peripheral and portal vein blood samples in 41 patients undergoing upfront surgery for PDAC (Tien *et al*., 2016) (Table 1). CTCs were detected (CellSearch^®^) in 39% of peripheral and 58.5% of portal vein blood samples. The presence of CTCs in the portal blood was a predictive factor of liver metastasis. The short follow‐up of this study (only 1 year after surgery) did not allow assessing the OS and progression‐free survival (PFS). In 14 patients with borderline resectable (*n* = 7) or metastatic PDAC (*n* = 7), CTCs (CellSearch^®^) were identified in 21% of peripheral blood samples and in 100% of portal samples, drawn by preoperative endoscopic ultrasound‐guided fine‐needle aspiration (EUS‐FNA) performed for diagnostic and staging purposes (Catenacci *et al*., 2015). Moreover, the absolute CTC numbers were higher in the portal blood samples (83.2/7.5mL versus 0.4/7.5mL in the peripheral blood samples). No correlation with OS or PFS was reported. The authors also evaluated the suitability of portal vein CTCs for gene expression studies. They found that downregulation of tumor‐suppressor genes had a strong prognostic value and could be used to stratify patients eligible for surgery, according to the relapse risk. Similarly, in 29 patients with locally advanced and metastatic tumors (Liu *et al*., 2018) (Table 1), CTCs were detected in 100% of the portal blood samples (obtained by ultrasonography‐guided transhepatic puncture) and in 54% of the peripheral blood samples, with a higher CTC count in portal than in peripheral blood (282/7.5mL versus 21/7.5mL). Moreover, the CTC count was correlated with liver metastases, and patients with a portal vein CTC count higher than 150/7.5mL had shorter OS. Finally, in this study portal vein CTCs were cultured ex vivo to test the response to several common chemotherapies. CTCs were highly resistant to gemcitabine and other standard clinical regimens such as 5‐FU and oxaliplatin. Moreover, CTC viability was reduced by deltarasin (Liu *et al*., 2018). This suggests that *ex vivo* CTC culture might be a valuable tool for choosing the best therapy for each patient.

**Table 1:** Comparison of CTC detection in peripheral and portal venous samples in patients with pancreatic ductal adenocarcinoma

Number of patients	CTC enrichment/detection methods	CTC count in peripheral blood	CTC count in portal blood	CTC detection rate in peripheral blood	CTC detection rate in portal blood	Prognostic value	References
29 Δθ	CD45^+^ leukocyte depletion/ClearBridge^®^	Mean 21 Med 9 Range 0–74	Mean 281 Med 174 Range 8–908	31%	100%	*n* > 150 OS 9.2 *n* < 150 OS 19.8	Liu *et al*. (2018)
20♦	EpCAM^+^ CTC selection/CellSearch^®^	Mean 0.25 Med 0 Range 0–2	Mean 6 Med 0 Range 0–103	20%	45%	Positive for CTCs: OS 23.1 months Negative for CTCs: 26.2 months	Bissolati *et al*. (2015)
41♦	EpCAM^+^ CTC selection/Immunocytochemistry	Mean 71 Med 40 Range 14–414	Mean 230 Med 60 Range 14–3579	39%	58.5% **∑**	Correlation between metastatic disease and CTC detection in portal blood	Tien *et al*. (2016)
14*Δ	EpCAM^+^ CTC selection/CellSearch^®^	Mean 0.7 Med 0 Range 0–7	Mean 125 Med 68 Range 1–516	21%	100%	NA	Catenacci *et al*. (2015)

CTC, circulating tumor cell; EpCAM, epithelial cell adhesion molecule; PDAC, pancreatic ductal adenocarcinoma; ICC, immunocytochemistry; PCR, polymerase chain reaction; NA, not applicable; NS, not significant; OS, overall survival; PFS, progression‐free survival; SD, standard deviation; Med, median.

Tumor stage in the studied population: ♦ resectable, * borderline, and Δ metastatic/locally advanced; θ neoadjuvant treatment before blood sampling.

∑: statistically significant difference between portal and peripheral samples

In these studies, CTC detection rate in peripheral blood was similar to what was previously reported (around 50%, as in Tables S1, 1), whereas in portal blood, it was on average 75%, for all tumor stages.

More studies with larger cohorts are needed to determine the value of this approach at early disease stages, particularly in patients with upfront resectable tumors. Interestingly, all studies found a correlation between liver metastases and portal CTCs, including in cohorts with resectable tumors. This suggests that CTC analysis in portal blood samples collected, for instance, during preoperative EUS‐FNA could be used to better select patients for surgery, especially patients with undetectable micrometastases. Indeed, echo‐endoscopy with puncture is now the gold standard for histologic proof and formal diagnosis, but has some limitations. It carries variable negative predictive value, because the endoscopic ultrasound‐guided fine‐needle aspiration biopsy yield and the pathological analysis are largely operator‐dependent. It is invasive and with high risk of morbidity, with possible induction of acute iatrogenic pancreatitis, sometimes compromising surgical management (Storm and Lee, 2016). CTC detection could contribute to decision‐making, particularly for triggering neoadjuvant and adjuvant treatment (Table 6).

Taken together, the results of these pilot studies suggest that liquid biopsy in the portal vein may help improving pancreatic cancer prognosis evaluation, and could be associated with tumor sampling during EUS‐FNA to improve PDAC management. For instance, CTC detection could be used as a companion diagnostic tool for the molecular/genetic analysis of cancer cells in patients with indication for neoadjuvant therapy. Indeed, preliminary data showed that CTCs could be useful to stratify patients and adjust the therapeutic options according to the cancer molecular characteristics (Soler *et al*., 2017).

Finally, functional testing of CTCs, such as detection/quantification of epithelial‐specific secreted factors by isolated cells, is still in the early days, but patient management might benefit from such approaches in the future (Table 6).

## 5 Colorectal cancer

CRC is the third most common cancer in both sexes. The 5‐year OS reaches almost 60%. About 50% of patients will develop metastatic disease that accounts for the majority of deaths (de Haas *et al*., 2011). After curative resection, approximately 30% of patients who develop metastases eventually die of metastatic disease. Although diagnosis of CRC by colonoscopy is routinely available, good prognostic markers to stratify patients according to the metastasis risk are still missing. It has been shown that CTC detection in peripheral blood is a good biomarker for poor prognosis such as PFS and OS in patients with metastatic CRC. Therefore, it could contribute to better tailor the patient general care. However, differently from breast and prostate tumors, CTC release in the peripheral blood by CRC is a rare event, and consequently, their detection is difficult in the clinical practice (less than 60% of positive patients for CTC detection rate) (Alix‐Panabières and Pantel, 2014; Tan and Hao, 2018). PCR‐based CTC detection methods did not improve sensitivity (Table S1). CTCs shed by CRC are disseminated via the mesenteric venous system that drains in the portal vein. The liver serves as a filter that retains many CTCs, including metastasis‐initiating cells at the origin of liver metastases (Denève *et al*., 2013). Nevertheless, other CTC subpopulations can pass through this organ to reach the peripheral blood (Figure 1). Denève *et al*. showed a decreasing mesenterico‐peripheral gradient of CTCs, with the liver as a frequent organ to accommodate distant metastases in CRC (Denève *et al*., 2013). Moreover, a study showed that immediately after tumor resection, CTC numbers decreased in the peripheral blood and in the local main vasculature of the tumor (Jiao *et al*., 2009) (Table 2). This study did not specify the percentage of patients with CTCs detected in the systemic circulation compared with the portal circulation, but the median CTC number before surgery, although very low in general, was higher in the portal circulation and hepatic vein. Moreover, CTC detection rate in the hepatic vein was lower than in the portal vein (17.5% versus 35%, respectively (Rahbari *et al*., 2012)), underlining the importance of the puncture site for CTC detection. In cohorts that included only patients with metastatic CRC, CTC detection rate was similar in peripheral blood and hepatic vein (46% and 54%, respectively), as well as the median CTC count (1 versus 2.5, respectively) (Connor *et al*., 2016) (Table 2). OS and PFS were worse in patients with CTC counts >3, suggesting that CTC detection and number could have a prognostic value in patients with metastatic CRC. When the tested population included only 20% of patients with metastatic diseases, CTC detection rate in mesenteric blood was almost twice higher than in peripheral blood (CellSearch^®^, 55.9% versus 29%, respectively). However, the small number of patients did not allow testing the correlation between CTC number and prognosis (Denève *et al*., 2013). This study also showed that using an EpCAM‐independent CTC enrichment method followed by the functional EPISPOT assay significantly increased CTC detection rate to 55.4% in peripheral blood, and only slightly (to 65.9%) in mesenteric blood samples. CTC number was significantly higher in mesenteric blood than peripheral blood samples, and more CTCs were detected with the EPISPOT assay than the CellSearch^®^ system. CTC detection (both methods) inversely correlated with the presence of lymphatic emboli, and only the EPISPOT results correlated with the primary CRC grading. Finally, cancer‐related survival was worse in patients without metastases but with more than 27 CTCs/15 mL of blood (only with the EPISPOT assay) (Table 2).

**Table 2:** Comparison of CTC detection in portal/mesenteric/hepatic vein and peripheral blood samples in patients with colorectal adenocarcinoma

Number of patients	CTC enrichment/detection methods	CTC count in peripheral blood	CTC count in portal blood	CTC count in hepatic vein/central vein	CTC detection rate in peripheral blood	CTC detectionrate in portal blood	CTC detection rate in hepatic vein/central vein (vena cava)	References
80Δθ	EpCAM^+^ CTC selection/CellSearch^®^	NA	Mean 1.5 Med 0 Range 0–32	Mean 0.3 Med 0 Range 0–5	NA	35% ∑	17.5% (via central line)	Rahbari *et al*. (2012)
75Δ	CD45^+^ leukocyte depletion EPISPOT ^®^/EpCAM^+^ selection/CellSearch^®^	EPISPOT^®^ Med 1.2 Range 0–92 CellSearch^®^ Med 0 Range 0–142	EPISPOT^®^ Med 4 Range 0–247 CellSearch^®^ Med 2.7 Range 0–286	NA	EPISPOT 55.4% CellSearch^®^ 29%	EPISPOT 65.9% ∑ CellSearch^®^ 55.9% ∑	NA	Denève *et al*. (2013)
29Δ	EpCAM^+^ CTC selection/CellSearch^®^	Open resection: Arterial Mean 1.82 Med 1 Range 0–6 Venous Mean 1.45 Med 1 Range 0–3	Open resection: Mean 1.5 Med 0 Range 0–32	Open resection: Mean 126 Med 87 Range 0–500	Open resection: Mean 174 Med 174 Range 0–500	NA	NA	Jiao *et al*. (2009)
31	EpCAM^+^ CTC selection/CellSearch^®^	**NA**	NA	NA	17%	72% ∑	NA	Wind *et al*. (2009)
63Δθ	EpCAM^+^ CTC selection/CellSearch^®^	Med 1 Interquartile range : 0–4		Med 2.5 Interquartile range :1–8	46%	NA	54% HV>3 ↓OS Multivariate analysis	Connor *et al*. (2016)

Tumor stage of the studied population: Δ metastatic; θ neoadjuvant treatment before blood sampling.

Prognostic value not evaluated except for [46]

∑: statistically significant difference between portal and peripheral samples

In conclusion, although CRC‐related CTCs are rare, many groups performed studies to test their diagnostic and prognostic relevance. Blood sampling closer to the tumor did not increase significantly CTC detection and numeration, including in the case of metastatic disease. It is possible that even close to the tumor, CRC‐released CTCs are rare. However, the EPISPOT assay allowed increasing CTC detection in mesenteric blood (Denève *et al*., 2013). CTC detection correlated with bad prognosis. Studies on larger cohorts are needed to test the value of CTC detection for CRC management.

New personalized treatment strategies for metastatic colorectal cancer require a better understanding of tumor biology and informative biomarkers (Gbolahan and O'Neil, 2019). CTCs could assist the implementation of current emerging therapeutic sequences (Table 6).

## 6 Hepatocellular carcinoma

Hepatocholangiocarcinoma is the sixth most prevalent cancer responsible for one‐third of all deaths by cancer (Ferlay *et al*., 2015). When diagnosed early enough, the 5‐year OS can reach 50%. Conversely, less than 10% of patients with stage IV disease survive the first year after diagnosis. Thus, tools for early screening are urgently needed, especially in high‐risk populations (patients with cirrhosis, hepatitis, and nonalcoholic steatosis hepatitis syndrome) who could greatly benefit from the available treatments in the case of early diagnosis. Currently, screening is based on the use HCC biomarkers, such as alpha‐fetoprotein (AFP) and des‐gamma‐carboxy prothrombin (DCP) (Tateishi *et al*., 2008). However, these markers show high false‐positive rates. Like for other cancers, CTC detection in peripheral blood is not sensitive enough to allow HCC diagnosis (Table S1). Nevertheless, CTC detection correlates with poor PFS and OS (Fan *et al*., 2015).

**Figure 2:**
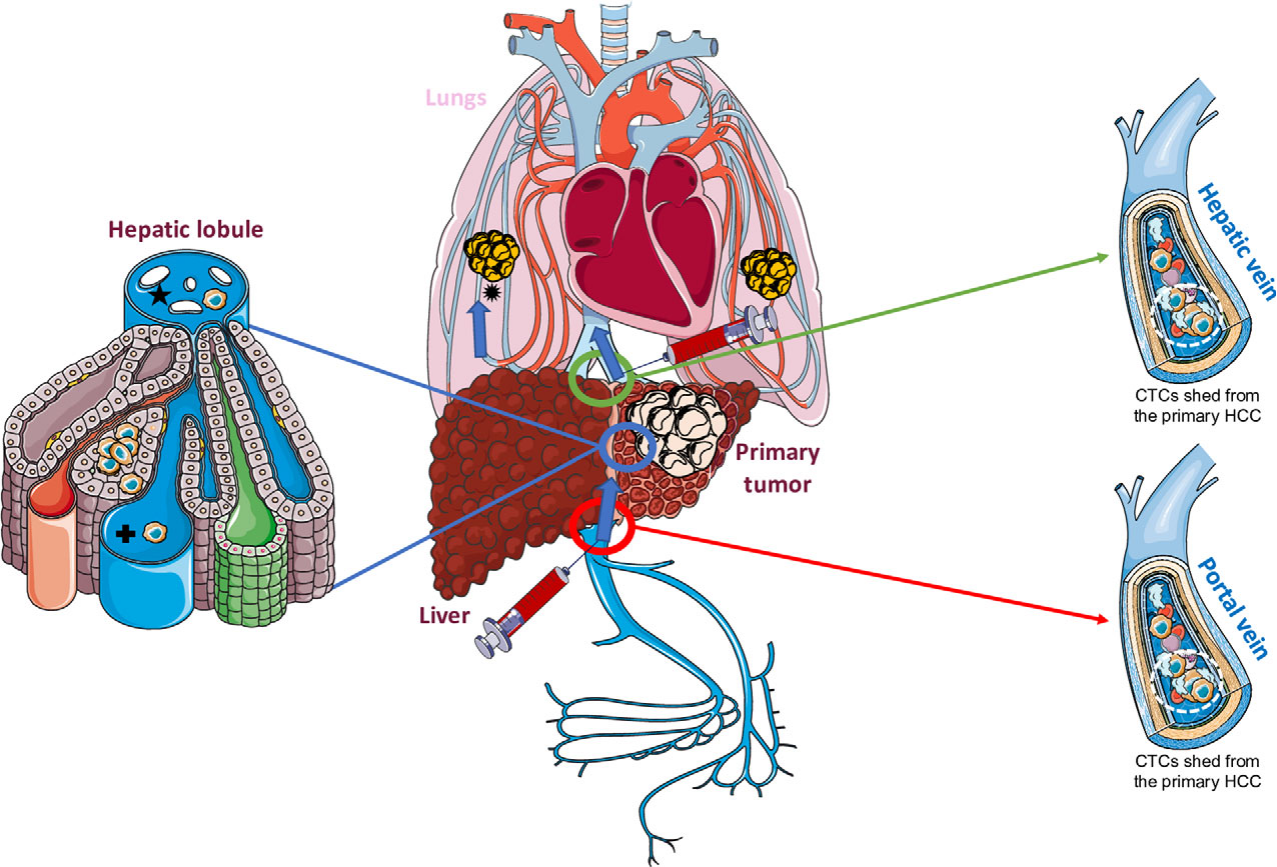
Detection of HCC‐derived CTCs in the hepatic and portal veins. The hepatic circulation is connected to the systemic circulation via three major vessels: the hepatic veins (green circle), which serve as the efferent pathway, and the hepatic artery and portal vein (red circle), which function as afferent vessels. HCC‐derived CTCs are released in the hepatic lobule (blue circle) in the portal branch (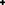) and in the central vein (★) that constitute the hepatic vein system draining into the inferior vena cava. They represent the main intrahepatic and pulmonary metastatic routes (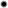). Blood sampling from the hepatic veins (green circle) could improve CTC detection.

Liver is connected with two major vascular systems: the hepatic veins that constitute the efferent pathway, and the hepatic artery and portal veins that compose the afferent pathway (Figure 2). CTCs from the primary liver tumor are first disseminated in microscopic portal vessels, and then in the centrolobular veins that drain into the main hepatic veins. CTCs can be detected also in the afferent system because HCC has a high propensity to colonize arterial vessels during neoangiogenesis (Figure 2) (Forner *et al*., 2012). We found only one study that compared CTC detection rates in function of the sampling site in patients with localized HCC (Table 3) (Sun *et al*., 2018). Detection rates in peripheral vein or artery blood samples (68% and 45%, respectively) were similar to previously published results (Tables 3, S1). As expected on the basis of the liver circulation, CTC recovery rate in portal blood and inferior vena cava did not increase compared with peripheral samples. Conversely, it was very high (80%) in the hepatic vein because this vessel drains all the microscopic lobular spaces that may receive CTCs. This study did not test the diagnostic/prognostic value of CTC detection in the different vessels. However, intrahepatic recurrence was strongly associated with CTC presence in peripheral blood samples (artery and vein) and with CTC micro‐emboli (or clusters). In addition, high detection rate of CTCs or clusters in the hepatic vein was associated with the presence of lung metastases.

**Table 3:** Blood sampling in different sites for CTC detection in patients with hepatocellular carcinoma (Sun *et al*., 2018)

Number of patients	CTC enrichment/Detection methods	Detection rate: pvCTC	Detection rate: paCTC	Detection rate: CTC in hepatic vein	Detection rate: CTC in portal blood	CTC recovery in inferior vena cava	Prognostic value
73*	EpCAM^+^ CTC selection/CellSearch^®^	68.49%	45.2%	80.82%	58.9%	39.72%	Intrahepatic recurrence: Univariate; PaCTC +PvCTC; Multivariate: PvCTC with CTM

CTM, circulating tumor micro‐emboli; paCTC, CTC in the peripheral artery blood; pvCTC, CTC in the peripheral venous blood.

*11% of patients had metastatic disease and none received neoadjuvant therapy

CTC count: Peripheral vein: median 2, range 0–26; Peripheral artery: median 0, range 0–11; Hepatic vein: median 6, range 0–31; Portal vein: median 1, range 0–8.

Differences in CTC detection rate between portal and peripheral venous samples statistically significant: ‐Peripheral vein vs hepatic vein; peripheral vein vs inferior vena cava; Peripheral artery vs hepatic vein; peripheral artery vs inferior vena cava; Hepatic vein vs portal vein.

In conclusion, CTC detection in the hepatic vein shows a strong prognostic value, both for disease recurrence and for disease dissemination (Fang *et al*., 2014). It would be interesting to test whether CTC presence, particularly in the hepatic vein, could be used to stratify patients eligible for adjuvant therapy. Moreover, future studies should assess whether CTC analysis could facilitate the individualized therapeutic decision‐making in HCC (Table 6).

## 7 Non‐small‐cell lung cancer

Lung and bronchus cancer remained the primary cause of death by cancer in 2017, representing around 25% of all deaths by cancer (Siegel *et al*., 2017). NSCLC accounts for 85% of all diagnosed lung cancers. This cancer is the most prevalent cancer in males and the third in females. Its 5‐year OS depends on the disease stage, going from 92% for stage IA1 to less than 1% for metastatic stage IV. Overall, the 5‐year survival rate is 18% (source: cancer.net). Even after surgery, tumor recurrence with distant metastases occurs in around 25% of patients, reaching approximately 29% in patients with stage I cancer (Goldstraw *et al*., 2016). Cytotoxic chemotherapy can slightly prolong survival in patients with tumor relapse. Indeed, it has been reported that the 5‐year survival rate improved only by 4% to 5% for patients with stage I–III NSCLC, and by only few months for patients with stage IV tumors, possibly because tumor recurrence is detected too late (Johnson *et al*., 2014).

**Figure 3:**
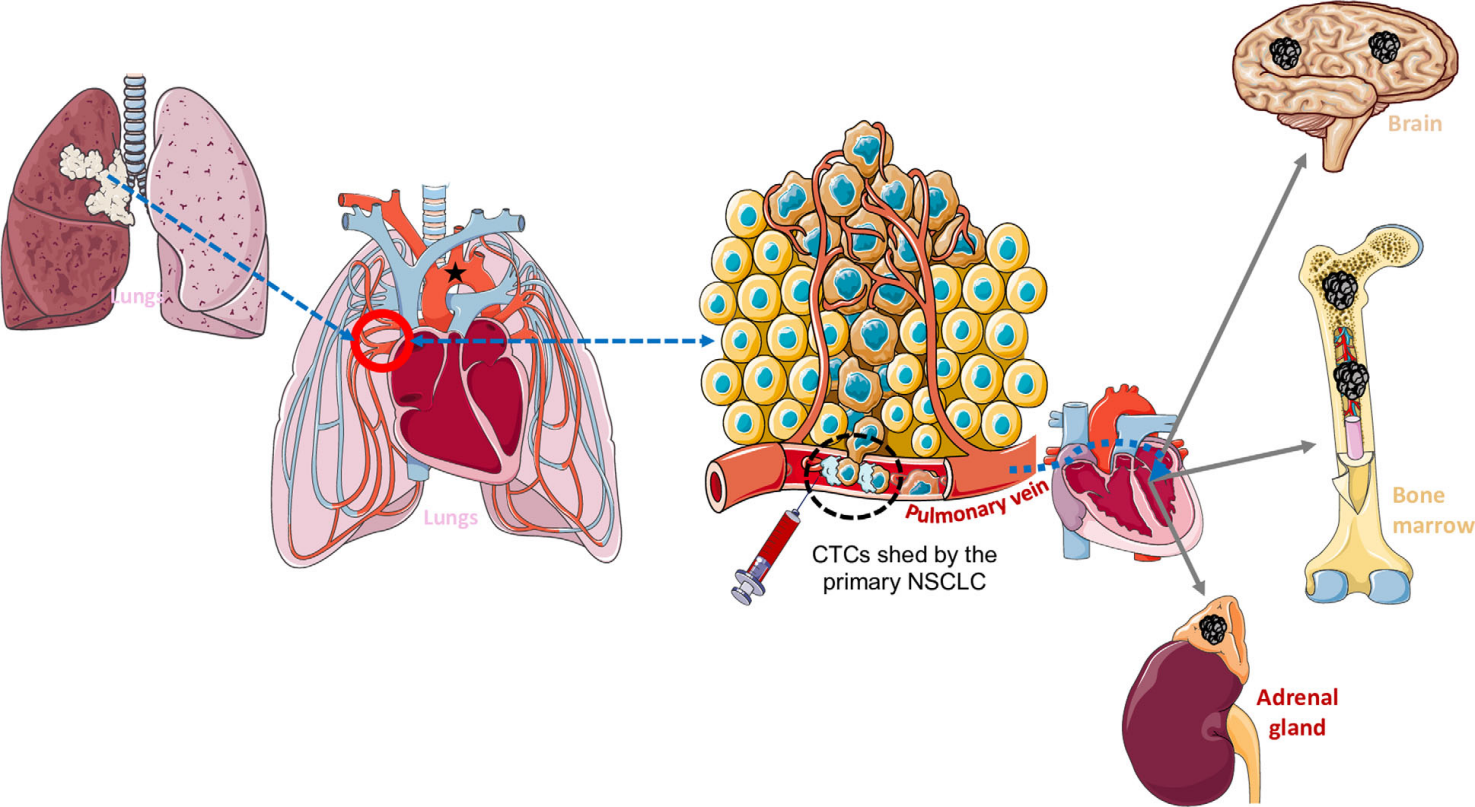
NSCLC‐derived CTC detection in the pulmonary vein. NSCLC metastatic sites are primarily bone marrow, brain, and adrenal gland. First, CTCs extravasate in the circulation via the pulmonary veins (black circle). Then, CTCs go into the systemic circulation toward the cerebral capillaries (via the branches of the aortic arch (★)) or the bone marrow sinusoids and other distant sites. The fenestrated structure of bone marrow sinusoid capillaries is permissive to cancer cell infiltration. Brain capillaries are more difficult to penetrate, due to the unique nature of the blood–brain barrier. Based on the features of the pulmonary circulation, CTCs could be retained in the pulmonary vein (black circle), offering an opportunity to increase their detection in blood samples collected from this vein during tumor resection, as already shown by Bernaudin *et al* 2005.

NSCLC‐derived CTCs disseminate first in the pulmonary vein (Figure 3) (Popper, 2016). Cancer cells follow the main bloodstream through the heart and join the systemic circulation where metastasis‐initiating cells can niche, mostly in the brain, bone marrow, adrenal gland, and liver. CTC detection in peripheral blood samples of patients with lung cancer has been evaluated in several studies (Table S1). Overall, the percentage of patients in whom CTC could be detected is quite low at all disease stages (around 53%) when using protein marker‐based CTC enrichment methods. CTC detection in patients with metastatic disease is more efficient when using PCR‐based methods (71%). Most studies reported a strong correlation between peripheral blood CTC detection and OS. Tumor recurrence also was associated with CTC detection (Gallo *et al*., 2017).

In 2005, CTCs were detected (RT‐PCR) for the first time in the pulmonary vein of patients with NSCLC (Bernaudin *et al*., 2005). Since then, many studies have compared CTC detection rate in peripheral blood and close‐to‐the‐tumor vessels. Like for PDAC and CRC, blood samples were collected close to the tumor drainage territory by puncturing the pulmonary vein. Most patients included in these studies had resectable tumors, and only a small percentage had metastatic disease. Overall, CTC detection rate in peripheral blood samples was similar to that of previously published works on peripheral blood only (Table 4; 45%, similar to the 53% in Table S1). Conversely, CTC detection rate in the pulmonary vein was about 91%. Of note, while the peripheral blood detection rates varied among reports (range 6.6–91.3%, mean 45.1% ± 34.1%), the pulmonary vein detection rate was quite reproducible (range 80–100%, mean 93.5% ± 7.3%). Similarly, the mean CTC count was higher in the pulmonary vein than in peripheral blood samples (544/7.5mL vs 3.1/7.5mL Table 4) (Reddy *et al*., 2016).

**Table 4:** CTC detection in pulmonary vein and peripheral vein samples in patients with non‐small‐cell lung cancer

Number of patients	CTC detection methods	CTC count in peripheral blood	CTC count in pulmonary vein blood	CTC detection rate in peripheral blood	CTC detection rate in pulmonary vein	Prognostic value	References
36	OncoBEAM^®^	Med 1.5 Range 0–15	Med 7.5 Range 0–10	69.4%	83.3% ∑	Shorter PFS associated with CTC clusters	Murlidhar *et al*. (2017)
30 Δ	Veridex^®^	Mean 0.8 Med 0 Range 0–16	Mean 1195 Med 81 Range 0–10034	16.7%	96.7%	NA	Okumura *et al*. (2009)
23	MACS+flow cytometry	Med 5 Interquartile range 3–9	Med 28 Interquartile range: 3–9	91.3%	95.7% ∑	High CTC count associated with lower PFS	Li *et al*. (2017)
10	ScreenCell^®^+immunochemistry analysis of 549 human lung cells	Mean 22 Range 0–100	Mean 65 Range 8–200	80%	100%	NS	Chudasama *et al*. (2017)
23 Δ	ScreenCell^®^+hematoxylin–eosin method	Cluster (CTC >4) *n* = 6 Single CTC *n* = 1	Cluster *n* = 15 Single CTC *n* = 4	30%	93%	NS	Sawabata *et al*. (2016)
30	CellSearch^®^	CTC ≥1/7.5mL *n* = 6 [1–4]	CTC ≥18/7.5mL *n* = 23	22.2%	100% ∑	High CTC count in peripheral blood associated with PFS and OS	Crosbie *et al*. (2016)
32 Δ	EpCAM‐based microfluidic chip	Mean 3.1 CTC/7.5mL	Mean 544 CTC/7.5mL ∑	NA	NA	NS	Reddy *et al*. (2016)
15 θ	EpCAM‐based microfluidic chip	NA	Mean 95.7 Range 0–855	6.6%	80%	Correlation with neoadjuvant therapyIT<SAPV CTC	Tarumi *et al*. (2013)

Tumor stage of the studied population: Δ metastatic; θ neoadjuvant treatment before blood sampling.

∑: statistically significant difference between pulmonary vein and peripheral samples.

IT, induction chemotherapy; PV CTC, pulmonary vein CTC; SA, surgery alone.

Results on the prognostic value of CTC presence in the pulmonary vein are heterogeneous. Some studies showed a correlation between CTCs and disease progression and OS (Crosbie *et al*., 2016; Li *et al*., 2017; Murlidhar *et al*., 2017; Tarumi *et al*., 2013). Other studies did not show a statistically significant association between CTC count or number of patients with CTCs in the pulmonary vein and OS or PFS(Chudasama *et al*., 2017; Hashimoto *et al*., 2014; Lv *et al*., 2018; Okumura *et al*., 2009; Reddy *et al*., 2016; Sawabata *et al*., 2016). However, a correlation between CTC levels and pathology, particularly tumor size, was described (Lv *et al*., 2018; Reddy *et al*., 2016). Sabawata's data suggested a shorter PFS when CTC clusters were present in the pulmonary vein, which was further studied by Murlidhar *et al*. who showed that the presence of clusters in the pulmonary vein was a factor of poor prognosis (shorter PFS) (Murlidhar *et al*., 2017). Additional studies did not evaluate the prognostic value of CTCs (Chudasama *et al*., 2017; Okumura *et al*., 2009) or did not include long‐term patient follow‐up (Hashimoto *et al*., 2014). Finally, one study showed that PFS and OS were associated with high CTC rate in peripheral blood, but not in the pulmonary vein blood (Crosbie *et al*., 2016).

The link between CTC detection in lung cancer and the surgery technique was assessed by few authors. Particularly, it was shown that surgical manipulation significantly increased CTC number in the pulmonary vein and that this was associated with lymphatic invasion and a significant reduction of PFS and OS (Hashimoto *et al*., 2014, 2018). A recent work suggested that intraoperative manipulation contributes to the hematogenous dissemination of tumorigenic CTCs and circulating tumor micro‐emboli (Table 5) (Lv *et al*., 2018). Similarly, it was reported that the CTC rate, including in the pulmonary vein, increases after endoscopic biopsy (Reddy *et al*., 2016). These data suggest that the pulmonary veins should be ligated before tumor mobilization to minimize tumor cell dissemination.

**Table 5:** CTC count after tumor mobilization in pulmonary vein and peripheral vein samples in patients with non‐small lung cancer

Number of patients	CTC detection methods	CTC count in pulmonary vein	CTC detection rate in peripheral blood	CTC detection rate in pulmonary vein	Prognostic value	References
30 Δ	CellSearch^®^	Med 60	6.7%	73.3%	NA	Hashimoto *et al*. (2014)
30	CellSearch^®^	Increase ΔCTC	No sample	80%	PFS OS metastasis correlated with ΔCTC	Hashimoto *et al*. (2018)
32	CellSearch^®^	Mean 617 Med 18 Range 1–8000	25%	90.6% ∑	NS	Lv *et al*. (2018)

Tumor stage of the studied population: Δ metastatic; θ neoadjuvant treatment before blood sampling

∑: statistically significant difference between pulmonary vein and peripheral samples

IT, induction chemotherapy; PV CTC, pulmonary vein CTC; SA, surgery alone.

Taken together, these results show that pulmonary vein puncture greatly increases the chances to detect CTCs originating from lung tumors, but the prognostic value for disease recurrence needs additional investigations with better categorization of the disease stages. Moreover, tumor cell dissemination during surgical procedure deserves to be better characterized. Nevertheless, CTC detection and characterization could help to obtain new insights into the tumor cell molecular features and design therapeutic strategies in NSCLC (Table 6).

**Table 6:** Tumor‐proximal *liquid biopsy*: perspective and potential clinical applications

Cancer types	Diagnosis (samples during the diagnostic assessment)		Prognosis (samples during surgery)		Monitoring (postoperative recurrence—MRD)	
	Sampling technique	Potential clinical application	Sampling technique	Potential clinical application	Sampling technique	Potential clinical application
PDAC	Echo‐guided portal puncture (EUS‐guided and external ultrasound‐guided portal vein puncture)	Companion diagnostic test; Decision algorithm for neoadjuvant treatment	Direct portal vein puncture	Decision algorithm for adjuvant chemotherapy	External ultrasound‐guided portal vein puncture	Confirmed disease relapse and monitoring metastatic disease
CRC	External ultrasound‐guided portal vein puncture	Companion test; Adapt therapeutic sequences	Direct portal vein puncture	Decision for adjuvant chemotherapy	External ultrasound‐guided portal vein puncture	Confirmed disease relapse and adapt personalized treatment
HCC	External ultrasound‐guided portal vein puncture and hepatic vein sampling (transjugular)	Staging and treatment strategy (i.e., ablation, resection, chemoembolization)	Direct portal and hepatic vein puncture	Decision for adjuvant chemotherapy	External ultrasound‐guided portal vein puncture and hepatic vein sampling (transjugular)	Staging and treatment strategy (i.e., ablation, resection, chemoembolization, liver transplantation)
NSCLC	Endovascular procedure	Companion diagnostic test; Staging and treatment strategy	Direct pulmonary vein puncture (before and after tumor mobilization)	Decision for adjuvant therapy	Endovascular procedure	Confirmed disease relapse and monitoring metastatic disease and adapt treatment

CRC, colorectal cancer; EUS, endoscopic ultrasound; HCC, Hepatocellular carcinoma; MRD, minimal residual disease; NSCLC, non‐small‐cell lung cancer; PDAC, pancreatic ductal adenocarcinoma.

## 8 Future directions

Analysis of the results obtained in different cancer types with similar approaches suggests that compared with peripheral CTC detection alone, combining CTC detection in the tumor‐draining vein and peripheral blood at the time of surgery or by ultrasonography‐guided puncture could improve the identification of patients at higher risk for cancer recurrence. Alternatively, diagnostic leukapheresis (DLA), a procedure recently introduced by Stoecklein's group to screen liters of blood (Andree *et al*., 2018; Fischer *et al*., 2013), could improve the chance of CTC recovery. Indeed, processing DLA products using CellSearch^®^ increased CTC yields up to 32‐fold.

Importantly, feasibility of portal vein puncture by ultrasonography‐guided puncture has already been successfully tested twice, allowing for the detection of CTCs for 100% of PDAC patients (Table 1 (Catenacci *et al*., 2015; Liu *et al*., 2018)). This approach is worth trying in HCC, since the only study testing CTC detection in the portal vein was promising for both CTC retrieval and prognostic performances (Table 3 (Sun *et al*., 2018)). For CRC, as CTC yields were not increased in mesenteric or portal vein, prognostic value was not tested in most of the studies. Thus, it is too early to recommend portal vein ultrasonography‐guided puncture in CRC patients. By contrast, pulmonary vein puncture repeatedly increased chances of CTC detection and carried high prognostic value (Tables 4 and 5). However, besides surgery, it is feasible to sample the pulmonary vein using endovascular procedure (Haïssaguerre *et al*., 1998).

Moreover, additional studies on CTC detection in vessels close to the primary tumors are needed, particularly to obtain crucial information on the tumor biology and the metastatic cascade by genomic analysis of isolated single CTC. Indeed, we need to learn more on CTC heterogeneity during their journey in the bloodstream, and the selection of CTC subclones through specific filtrating organs (e.g., the liver) (Joosse *et al*., 2018). This particular aspect could be evaluated by comparing peripheral CTCs versus tumor vicinity CTCs by single‐cell analysis. Based on the hypothesis that CTCs represent cells at the origin of metastases, these cells could also predict the genetic landscape of the metastatic tumors. For example, in cancers that carry multiple genetic mutations, these alterations may not be homogeneously distributed and the tumor biopsy may not show all the mutations. Thus, CTC analysis can contribute to the genetic/molecular characterization of the tumor for prognostic/therapy stratification purposes (Table 6), and also to the discovery of new biomarkers.

## Conflict of interest

The authors declare no conflict of interest.

## Supporting information


**Table S1** Main studies for CTC detection in the peripheral blood of patients with PDAC, CRC, NSCLC, and HCC.
