# Supplementary material for: Tumor‐proximal liquid biopsy to improve diagnostic and prognostic performances of circulating tumor cells
Source: Mol Oncol. 2019 Jul 25;13(9):1811–26. doi: 10.1002/1878-0261.12534 (PMC6717761; doi:10.1002/1878-0261.12534)
Supplement: Supplementary file 1 — Table S1 Main studies for CTC detection in the peripheral blood of patients with PDAC, CRC, NSCLC, and HCC. [file MOL2-13-1811-s001.docx]

**Supplemental data**

**Supplemental Table 1.** Main studies for CTC detection in the peripheral blood of patients with PDAC, CRC, NSCLC and HCC.

| Cancer types | Methods | Studies(n) | Nb Patients (with cancer) | Disease stage | Results  Mean%+/- SD | References |
| --- | --- | --- | --- | --- | --- | --- |
| PDAC | CellSearch®/ICC | 4 | 194 | All stages | 49.6+/-29.9 | (Kurihara et al. 2008; Bidard et al. 2013; Earl et al. 2015; Khoja et al. 2012) |
|  | Filtration | 4 | 180 | All stage | 72.1+/-  18.3 | (Iwanicki-Caron et al. 2013; Cauley et al. 2015; Bobek et al. 2014; Kulemann et al. 2015) |
|  | PCR-based | 7 | 344 | All stage | 68.5+/-21.7 | (Funaki et al. 1996; Miyazono et al. 1999; Chausovsky et al. 1999; Zhang et al. 2005; Soeth et al. 2005; Ishizone et al. 2006; Hoffmann et al. 2007) |
| CCR | Cellsearch®/ICC | 11 | 1893 | Metastatic | 35.63+/-  19.85 | (S. J. Cohen et al. 2009; Schoppmeyer et al. 2006; Steven J. Cohen et al. 2006; Hiraiwa et al. 2008; Königsberg et al. 2010; Matsusaka et al. 2011; Papavasiliou et al. 2010; Tol et al. 2010; Aggarwal et al. 2013; Seeberg et al. 2015; Sastre et al. 2013) |
|  |  | 3 | 878 | Non-metastatic | 23.33+/-  15.27 | (Bork et al. 2015; Sotelo et al. 2015; P. Gazzaniga et al. 2013) |
|  | PCR-Based | 7 | 309 | Metastatic | 43.28+/-  17.43 | (Vlems et al. 2003; Paola Gazzaniga et al. 2010; Rahbari et al. 2011; Koch et al. 2005; Staritz et al. 2004; Wyld et al. 1998; Yen et al. 2009) |
| HCC | Cellsearch®/ICC | 12 | 963 | NA | 57.83+/-  18.03 | (Kelley et al. 2015; Vona et al. 2004; S. Liu et al. 2013; Fan et al. 2011; W. Xu et al. 2011; Y.-M. Li et al. 2013; Schulze et al. 2013; Sun et al. 2013; Fang et al. 2014; Guo et al. 2014; Jun Li et al. 2014; Mu et al. 2014) |
|  | PCR-Based | 8 | 881 | NA | 43.75+/-  19.06 | (Matsumura et al. 1999; Cillo et al. 2004; Jeng, Sheen, et Tsai 2004; Witzigmann et al. 2002; Mou et al. 2002; Yao et al. 2013; Choi et al. 2015; Kong et al. 2009) |
| NSCLC | Cellsearch®/ICC | 4 | 483 | Non-metastatic | 53.5+/-  10.63 | (Sawabata et al. 2007; Hofman, Bonnetaud, et al. 2011; Hofman, Ilie, et al. 2011; Bayarri-Lara et al. 2016) |
|  |  | 6 | 312 | Metastatic | 53.33+/-  24.4 | (Krebs et al. 2011; Muinelo-Romay et al. 2014; Y. H. Xu, Zhou, et Pan 2015; Juan et al. 2014; Punnoose et al. 2012; Isobe et al. 2012) |
|  | PCR-Based | 3 | 250 | Non-metastatic | 39.66+/-  19 | (Yamashita et al. 2002; Yoon et al. 2011; Jian Li et al. 2014) |
|  |  | 5 | 288 | Metastatic | 71.8+/-  7.15 | (Du et al. 2014; Nieva et al. 2012; L. Liu et al. 2008; Sher et al. 2005; Chen et al. 2007) |
